# Thirty Days Outcomes of Limb Salvage Surgery in Pediatric Patients Treated at a Tertiary Care Hospital

**DOI:** 10.7759/cureus.74672

**Published:** 2024-11-28

**Authors:** Muhammad Younus Khan Durrani, Usman Ali, Javeria Saeed, Masood Umer

**Affiliations:** 1 Orthopaedic Surgery, Aga Khan University Hospital, Karachi, PAK

**Keywords:** limb salvage surgery, low- and middle-income country, morbidity, mortality, musculoskeletal tumors, pediatric, pediatric orthopedic surgery, post-surgical complications, sarcomas

## Abstract

Background

Managing primary bone and soft tissue sarcomas in pediatric patients poses significant challenges, with surgical resection remaining essential for cure. While limb salvage surgery has emerged as the standard approach, concerns persist regarding post-operative complications. Our study aims to evaluate the 30-day morbidity and mortality of limb salvage surgery in pediatric tumor patients, bridging critical knowledge gaps and contributing to enhancing the standard of care in low- and middle-income countries (LMICs).

Materials and methods

A prospective cohort study was conducted on pediatric patients (aged 18 years or younger) who presented at a tertiary care hospital's orthopedics department and required limb salvage surgery due to various tumors from May 2021 to October 2023.

Results

Nineteen cases met the inclusion criteria. The average patient age was 12.2 years (SD = 4.71), with 5 (20.8%) females and 14 (79.2%) males. Bone tumors accounted for 12 (63.2%) cases, and soft tissue tumors for 7 (36.8%). Osteosarcoma was the most common tumor (36.8%), followed by Ewing Sarcoma (31.57%) and Synovial Sarcomas (15.8%). Free fibular grafts were used in 6 (31.6%) cases, and Mega prosthesis in 3 (15.8%). Overall, 26.3% of cases experienced post-surgical complications within 30 days, notably including surgical site infections (21.05%) and flap necrosis (10.53%). No significant differences in demographic and clinical variables were observed between patients with and without complications.

Conclusion

Our study highlights immediate post-operative outcomes and complications of limb salvage surgery in pediatric musculoskeletal tumor patients, particularly in LMIC settings. Despite advancements, early complications remain challenging, with nearly one-quarter of patients experiencing adverse events within 30 days. The prevalence of surgical site infections emphasizes the urgent need for improved infection control measures in LMICs.

## Introduction

Managing primary bone and soft tissue sarcomas in pediatric patients poses a formidable challenge in terms of treatment. Chemotherapy has substantially improved survival, but surgical resection remains essential for cure; hence, intricate surgical interventions for local control become imminent [1,2].

Advancements in chemotherapy protocols, implant technology, reconstructive methods, and surgical approaches have shifted the paradigm in adolescent tumor (sarcomas) treatment. Previously, radical measures such as amputation were predominant; however, limb salvage has now emerged as the standard approach [3]. This transition from traditional amputation to contemporary limb salvage surgery has significantly improved functional outcomes [1,4,5].

Despite these advancements, concerns persist regarding the post-operative complications associated with limb salvage procedures, potentially influencing the psychosocial aspect of children and parents, financial burden, adjuvant therapy timelines, and long-term oncologic outcomes [6]. In the past four decades, there has been an increasing number of limb cancer patients undergoing salvage compared to amputations in the prior era [7]. This shift can be attributed to ongoing research and the emergence of newer limb salvage options. Surgeons' ingenuity in addressing tumor defects and restoring functionality has significantly increased patient satisfaction. Moreover, studies indicate that limb salvage procedures yield comparable disease-free progression outcomes to amputation [7,8].

Research demonstrates that limb salvage surgery in pediatric patients with primary extremity musculoskeletal tumors offers superior overall survival (OS) and disease-specific survival (DSS) compared to amputation [9]. Limb salvage procedures, while offering significant advantages over amputation, can still pose complications. Early post-operative complications, ranging from 14% to 21.7%, are commonly observed, with surgical site infections, sepsis, and venous thromboembolism being predominant concerns [4,5,7,10]. Conversely, amputation cases in pediatric populations have been associated with higher rates of severe medical complications, including acute renal failure, cardiac arrest necessitating CPR, and myocardial infarction. Notably, approximately one in seven patients experience complications within the initial thirty days following surgery for primary bone and soft tissue sarcomas of the extremities [4,6].

Advancements in imaging techniques and reconstructive options significantly contribute to the success of limb salvage surgery by facilitating the restoration of functional limbs [11]. Establishing a multidisciplinary limb salvage team has demonstrated promising outcomes, correlating with improved one-year survival rates [1]. As a result, limb salvage has emerged as a pivotal area of interest, with efforts directed toward refining techniques to improve patient outcomes [7].

To our knowledge, there is a significant dearth of prospective studies examining pediatric limb salvage following musculoskeletal tumors, especially in low- and middle-income countries (LMICs). Therefore, our study aims to bridge this gap by evaluating the 30-day morbidity and mortality associated with limb salvage surgery in pediatric sarcoma patients treated at our center in an LMIC setting. Through this effort, we aspire to address critical knowledge gaps and contribute to enhancing the standard of care for pediatric oncology patients in LMICs.

## Materials and methods

This prospective cohort study was conducted at the orthopedics department of a major tertiary care hospital in the developing world. Appropriate approval from the Ethical Review Committee was obtained. The study period spanned from May 2021 to October 2023.

Pediatric patients (aged less than 18 years) presenting with types of sarcoma necessitating limb salvage surgery were included in the study. After all appropriate consents and assent for participation in the study, patients planned for wide-margin resection of musculoskeletal tumors were recruited upon admission. Exclusion criteria encompassed patients who had undergone index surgery outside the study hospital or had previously undergone index surgery.

Data collection commenced on the day of surgery and continued for 30 days post-operatively, with meticulous recording of complications. Patient demographics, prior comorbid conditions, tumor characteristics, surgical modalities, and complications occurring in the early post-operative period were extracted as variables of interest. The data were assessed for both local and systemic complications. Tumors were categorized into bone and soft tissue tumors, with the final histopathology reports determining tumor types. Additionally, the presence of grafts and flaps, which can complicate procedures and increase the likelihood of complications, was recorded and tabulated. The plastic surgery team predominantly carried out reconstruction procedures. The study has been reported in line with the reporting Of cohort studies in surgery (STROCSS) criteria [12].

All data collected was stored in a secure electronic file, accessible only to the research team. Access to this file was restricted to team members solely for the purpose of analysis, ensuring the protection and confidentiality of patient information.

Upon completion of data collection, the gathered data were compiled and analyzed using the Statistical Package for the Social Sciences (SPSS). Quantitative variables such as age were reported as mean and standard deviation, while qualitative variables such as gender and complications were presented as frequency and percentage. The association of qualitative variables with post-operative complications was assessed using the chi-square/Fisher exact test. Throughout the study, a p-value of <0.05 was considered significant.

## Results

A total of 19 pediatric patients were included in this prospective study. The mean age of the patients was 12.2 years (SD=4.71), ranging from 5 to 17 years. Among the patients, 79.2% were males (n=14) and 20.8% were females (n = 5). None of the patients had any comorbid conditions. Tumor characteristics varied, with the majority of cases involving bone tumors (63.2%, n=12), while the remaining cases were attributed to soft tissue tumors (36.8%, n=7). The most prevalent tumor type identified from histopathology reports was osteosarcoma, accounting for 42.1% of cases (n=8), followed by Ewing Sarcoma (31.5%, n=6), and Synovial Sarcoma (15.8%, n=3) (Table 1).

**Table 1:** Tumor types identified from histopathology reports

Tumor types	n	%
Osteosarcoma	8	42.10
Ewing Sarcoma	6	31.57
Synovial Sarcoma	3	15.79
Epithelioid Sarcoma - Spindle Cell Sarcoma	1	5.26
Myxoid Pleomorphic Liposarcoma	1	5.26

Surgical interventions encompassed a range of procedures, including wide-margin excision, flap reconstruction, and grafting techniques. Notably, 57.9% of cases (n=11) received some form of neoadjuvant therapy, predominantly chemotherapy.

Regarding post-operative complications, 14 (73.6%) patients experienced no complications, while 5 (26.4%) patients had post-operative complications (one patient experiencing more than one complication simultaneously). Surgical site infections (SSI) were present in four patients (21.05%), and 2 (10.53%) experienced flap necrosis, with 1 patient experiencing both SSI and flap necrosis. Management of the complications is listed in Table 2.

**Table 2:** Post-operative complications and treatment *1 patient had both flap necrosis and SSI as a complication

Complications	N (%)
Yes	5 (26.31)
No	14 (73.68)
Surgical Site Infection*	4 (21.05)
Flap Necrosis	2 (10.53)
Complications Treatment	-
Outpatient Antibiotics	3 (16.67)
Surgical debridement	1 (5.55)
Redo Surgery of the Flap + Graft	1 (5.55)

Additionally, our analysis of demographic and clinical variables revealed no significant differences in age (p=0.709), gender (p=0.709), tumor type (p=0.106), anatomical location of the tumor (p=0.709), or use of flap (p=0.106) between patients with and without complications. However, a trend was observed for the use of flap (p=0.106), neoadjuvant therapy (p=0.106), and tumor type (p=0.106), suggesting potential associations with post-operative outcomes. Table 3 gives a detailed outlook on the analysis.

**Table 3:** Demographic and clinical variables for post-operative complications Fisher's Exact Test was utilized to provide a p-value indicating whether the association for each variable is statistically significant (typically p<0.05) or not. (Test values cannot be generated while utilizing Fisher's Exact Test).

	Without complication frequency n=14(%)	With Post-operative complications, frequency n=5(%)	p-value
Age	10.86±4.51	14.40±2.80	
Gender			1.00
Male	10(71.43%)	4(80.0%)	
Female	4(28.57%)	1(20.0%)	
Type of tumor			0.106
Bone Tumor	7(50%)	5(100%)	
Soft tissue Tumor	7	0 (0%)	
Anatomical location of the tumor			1.00
Upper limb	4(28.57%)	1(20%)	
Lower limb	10(71.43%)	4(80%)	
Use of Flap			0.106
Yes	7(50.0.%)	5(100%)	
No	7(50.0%)	0(0%)	
Neoadjuvant therapy			0.106
Yes	7(50.0%)	5(100%)	
No	7(50.0%)	0(0%)	
Adjuvant chemotherapy			0.57
Yes	3(21.43%)	2(40%)	
No	11(78.57%)	3(60%)	
Radiotherapy			0.57
Yes	11(78.57%)	3(60%)	
No	3(21.43%)	2(40%)	

**Figure 1:**
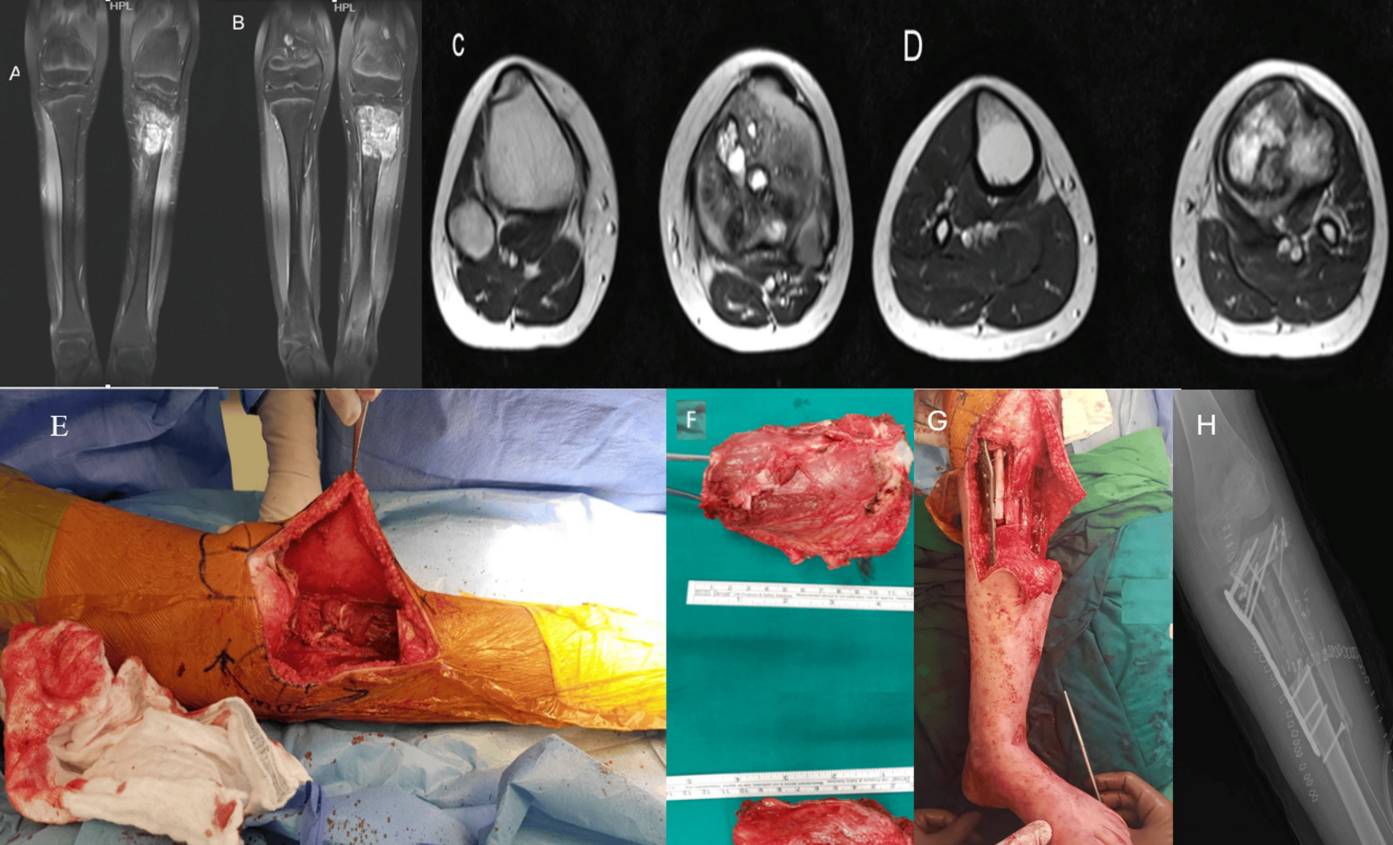
Clinical, imaging, and surgical sequence of a 13-year-old patient with osteoblastic osteosarcoma of the proximal tibia A 13-year-old girl presented with a complaint of swelling below her left knee, persisting for two months. Clinical examination revealed a tender, firm swelling approximately 3x3 cm in size. X-ray findings showed a lytic lesion involving the proximal tibia, raising suspicion of neoplastic involvement. A subsequent biopsy confirmed the diagnosis of osteoblastic osteosarcoma. Following the diagnosis, the patient underwent a course of neoadjuvant chemotherapy comprising Cisplatin, Adriamycin, Doxorubicin, and Methotrexate over six cycles spanning 10 weeks. Post-chemotherapy imaging studies, including MRI, were conducted (Fig A-D). The patient underwent a surgical procedure involving a wide-margin excision and an ipsilateral double-barrel pedicled fibular graft. Intraoperative pictures (Fig E-G) and post-operative X-rays (Fig H) were obtained.

**Figure 2:**
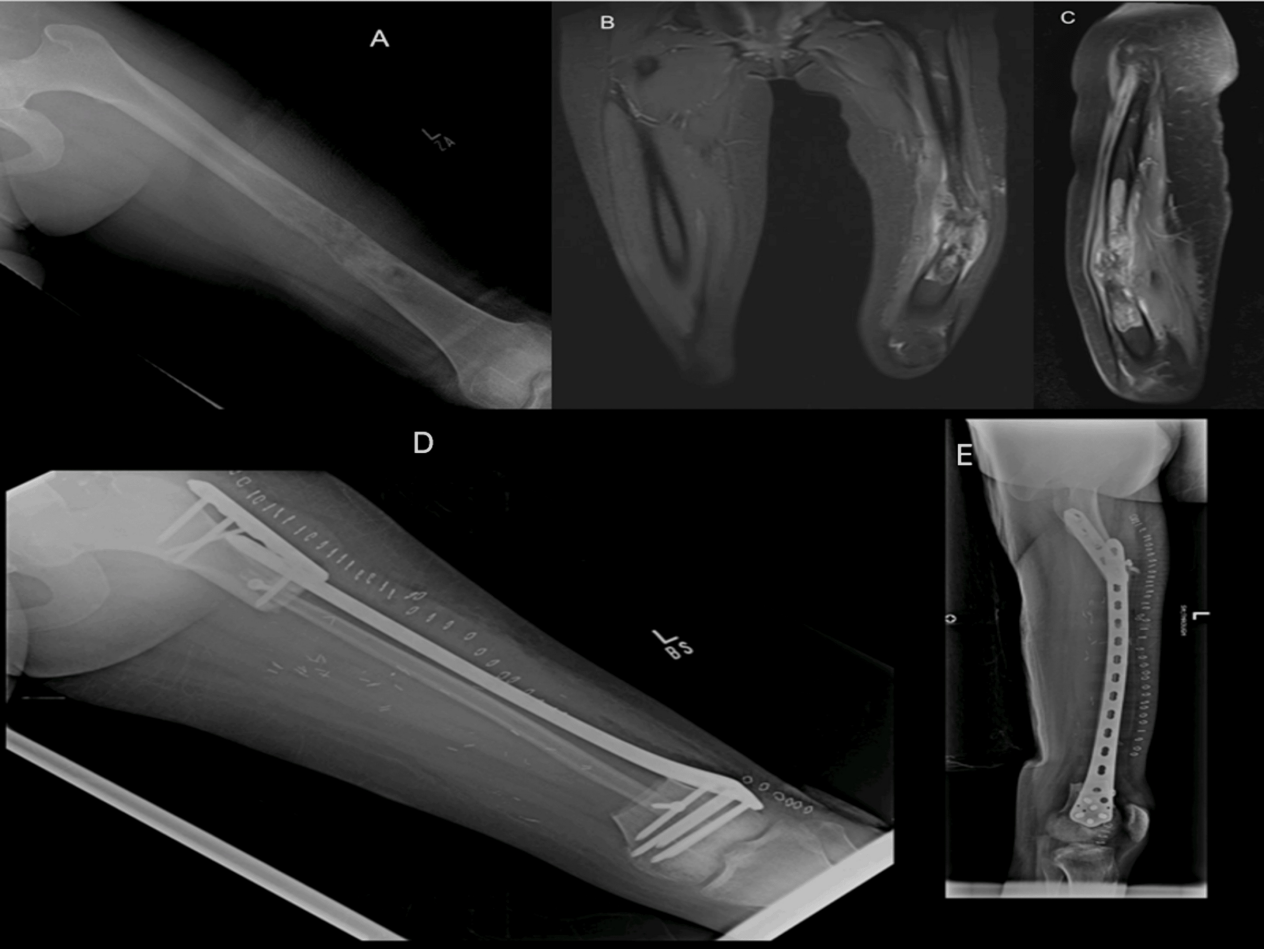
Diagnostic, surgical, and post-treatment imaging of a 13-year-old patient with Osteosarcoma of the left femur A 13-year-old girl presented with a two-year history of progressively worsening left leg pain and weight loss without neurological symptoms. A biopsy of her left femur confirmed the diagnosis of osteosarcoma. Following this diagnosis, she underwent neo-adjuvant chemotherapy according to the MAP protocol for two cycles. Subsequently, she underwent surgery involving excision of the tumor, vascularized free fibula grafting, and placement of a distal femur plate. • Figures A-C: Post-chemotherapy MRI images showing the response to treatment and planning for surgical intervention. • Figure D-E: Post-operative X-rays demonstrating the placement of the distal femur plate and the vascularized free fibula graft.

**Figure 3:**
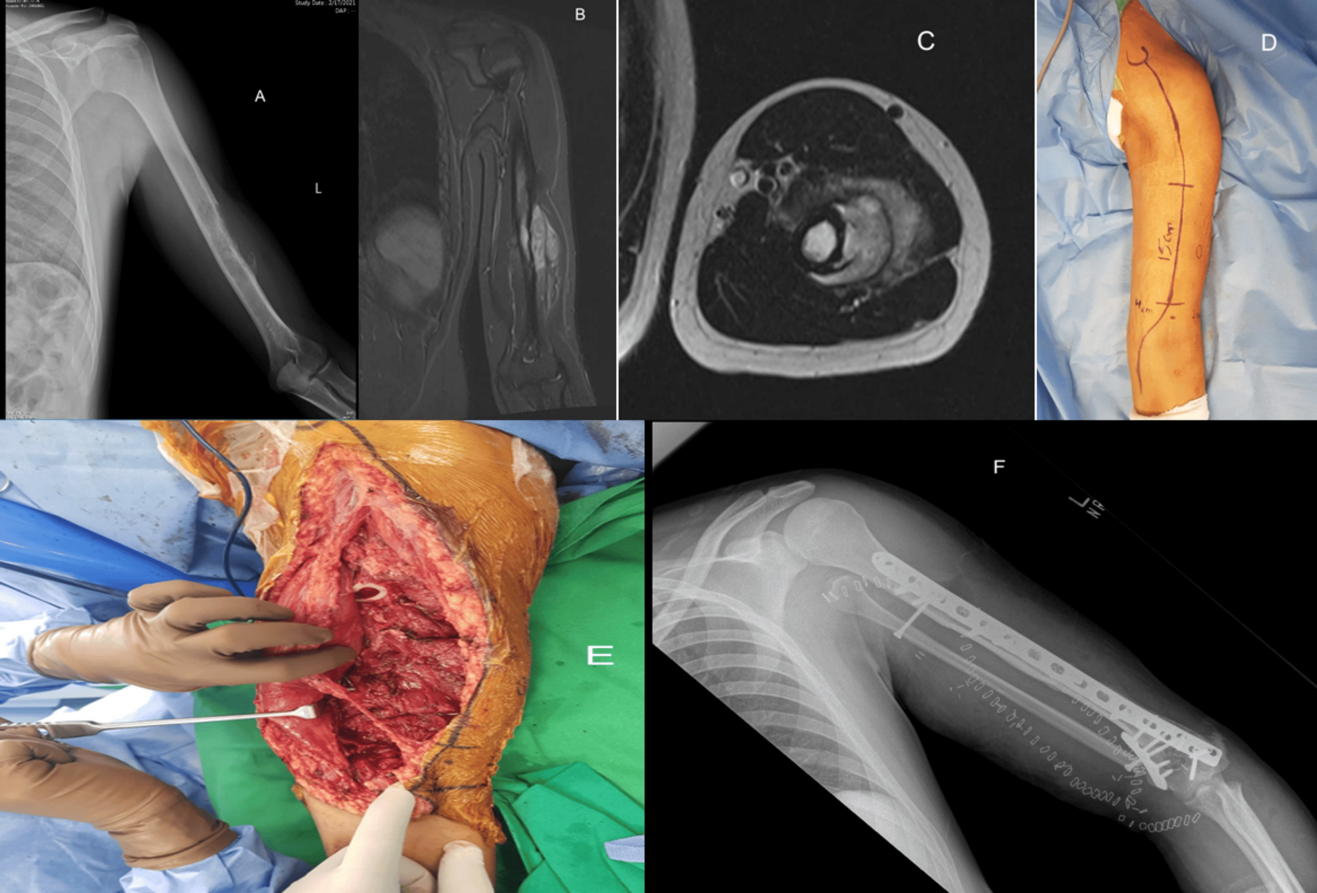
Imaging and surgical management of a 17-year-old patient with Ewing sarcoma of the left distal humerus A 17-year-old boy presented with a complaint of swelling in his left arm that had been present for four months, with no history of trauma. The swelling had been gradually increasing in size. A biopsy confirmed the diagnosis of Ewing sarcoma. The treatment involved a wide-margin excision of the left distal humerus, followed by reconstruction using a free fibular graft and autoclaved bone insertion. • Figures A-C: Preoperative X-rays and MRI scans showing the extent of the tumor and the surrounding anatomy. • Figures D-E: Intraoperative pictures illustrating the surgical procedure, including the excision and reconstruction process. • Figure F: Post-operative X-rays demonstrating the placement of the free fibular graft and autoclaved bone in the resected area.

Figures 1-3 provide detailed visual representations of three cases: A 13-year-old girl with osteosarcoma of the left femur, another 13-year-old girl with osteosarcoma of the left proximal tibia, and a 17-year-old boy with Ewing sarcoma of the left distal humerus. These figures illustrate the progression from diagnosis through surgical intervention and post-operative recovery, including preoperative imaging, intraoperative photographs, and post-operative outcomes.

## Discussion

The study analyzed the 30-day post-operative outcomes and complications following limb salvage surgery in pediatric patients with musculoskeletal tumors. With a cohort of 19 patients, predominantly male and receiving neoadjuvant chemotherapy, we observed a notable incidence of post-operative complications. Nearly a quarter of patients encountered adverse events within the initial 30-day post-surgery period. Notably, surgical site infections emerged as the predominant complication, requiring diverse interventions, including outpatient antibiotic therapy, surgical debridement, and flap redo procedures.

The patient demographics in this study revealed that individuals with soft tissue sarcomas were younger compared to those with bone sarcomas. Additionally, male gender was significantly more prevalent across the entire study population and within the subgroups. These demographic findings align with established epidemiological trends observed in childhood sarcomas [13].

Limb salvage procedures for musculoskeletal tumors in the pediatric population have shown significant advancements over the years, resulting in improved functional outcomes and quality of life [7,14]. However, early post-operative complications continue to pose a considerable burden. Our study revealed that nearly one-quarter of patients experienced post-operative complications, which exceeds the accepted rate for such surgeries. A study by Kathryn et al., involving 192 pediatric patients with similar pathologies, reported an overall complication rate of 8.9% [6]. Another study conducted by Yoichi et al. concluded an overall complication rate of 46% with a 31% rate of re-operation, which is significantly higher than our study. This can be attributed to the longer follow-up duration [15].

Conversely, Ziad et al. documented a postoperative complication rate of 21.7% in adult limb salvage surgeries conducted in low- and middle-income country (LMIC) settings [16]. The variance in complication rates may stem from diverse geographic and socioeconomic conditions among patients and disparities in available resources. LMICs, in particular, tend to exhibit significantly higher complication rates compared to developed nations, as concluded by Hasan et al. [17]. The high complication rates observed in our study may have been influenced by factors such as limited access to healthcare resources, variations in infection control practices, and differences in post-operative care.

In sarcoma patients, post-operative infectious complications remain a concern. Kathryn et al. [6] reported a lower overall complication rate of 8.9%, contrasting our 26.3%. Variations in infection rates may stem from geographic and socioeconomic factors, with developing countries like ours reporting higher rates due to resource limitations. Adult studies similarly found infection rates ranging from 1.7% to 7.0%. Yoichi et al. [15] also identified the infection as a common complication, with wound dehiscence noted as frequent, likely due to longer follow-up periods allowing for such complications to manifest. Our study did not address long-term complications such as prosthetic loosening and wound dehiscence due to its shorter follow-up period.

Patient characteristics such as age, gender, prior comorbidities, tumor type and location, surgical procedure, and neoadjuvant or adjuvant therapies are often considered potential risk factors for post-operative complications. Prior studies have suggested a correlation between patient demographics and increased rates of wound complications. However, our study did not find a significant association between patient demographics and the incidence of post-operative complications (p-value > 0.005).

The anatomical location of the tumor is widely recognized as a significant risk factor for post-operative wound complications. Variations in anatomy, particularly proximity to critical neurovascular structures and joint spaces, pose challenges during surgical resection. Korah et al. identified the anatomical location of the tumor as a primary risk factor for post-operative wound complications, particularly noting a higher occurrence of sarcomas situated in the lower extremities [18]. Although our study results coincide with this pattern, with the majority of complications observed in lower limb cases, we were unable to establish statistically significant associations, potentially due to the limited size of our patient cohort.

Another significant factor potentially influencing post-operative outcomes is the type of lost tissue. Factors such as the availability and size of transferable local tissue, vascular inflow, and the chosen method of bony reconstruction play crucial roles [19]. Our findings reflect this, as post-operative complications were observed more frequently in patients with bony tumors compared to those with soft tissue sarcomas. However, statistical significance was not achieved in this regard.

While neoadjuvant chemotherapy increases the risk of infection by weakening the immune system, it is commonly used as an adjunct to surgery for high-grade tumors. [20] Despite its widespread use, neoadjuvant chemotherapy remains a topic of debate among clinicians. A study by Gronchi et al. highlighted that the concomitant administration of chemotherapy and radiation therapy significantly increases the risk of high-grade thrombocytopenia and post-operative wound complications [21]. However, our findings, consistent with those observed by Ziad et al., did not show a statistically significant association between neoadjuvant therapy and the occurrence of complications [16].

## Conclusions

In conclusion, our study sheds light on the immediate post-operative outcomes and complications following limb salvage surgery in pediatric patients with musculoskeletal tumors, particularly in the unique context of an LMIC setting. Despite significant advancements in limb salvage techniques, early post-operative complications remain a formidable challenge, with nearly one-quarter of patients experiencing adverse events within the initial 30-day period. The prevalence of surgical site infections underscores the pressing need for improved infection control measures in LMICs, where resource constraints may exacerbate the risk of post-operative infections.

While our study provides valuable insights, its limitations, including small sample size and short follow-up duration, emphasize the necessity for further prospective research with larger cohorts and longer follow-up periods to comprehensively understand limb salvage surgery outcomes in this vulnerable population. As a pilot study, we advocate for heightened awareness within the medical and surgical communities regarding the significant concern posed by infection rates in pediatric limb salvage surgery. Addressing this issue demands enhanced perioperative antibiotic coverage, stringent infection control protocols, and the undertaking of prospective studies with larger sample sizes to validate our findings and inform evidence-based practices.
